# Relationship between hyposalivation and oxidative stress in aging mice

**DOI:** 10.3164/jcbn.16-79

**Published:** 2017-06-23

**Authors:** Yoshitaka Yamauchi, Tomonori Matsuno, Kazuhiko Omata, Tazuko Satoh

**Affiliations:** 1 Department of Oral and Maxillofacial Surgery, The Nippon Dental University School of Life Dentistry at Tokyo, 1-9-20 Fujimi, Chiyoda-ku, Tokyo 102-8159, Japan

**Keywords:** aging, hyposalivation, oxidative stress, salivary gland

## Abstract

The increase in oxidative stress that accompanies aging has been implicated in the abnormal advance of aging and in the onset of various systemic diseases. However, the details of what effects the increase in oxidative stress that accompanies aging has on saliva secretion are not known. In this study, naturally aging mice were used to examine the stimulated whole saliva flow rate, saliva and serum oxidative stress, antioxidant level, submandibular gland H-E staining, and immunofluorescence staining to investigate the effect of aging on the volume of saliva secretion and the relationship with oxidative stress, as well as the effect of aging on the structure of salivary gland tissue. The stimulated whole saliva flow rate decreased significantly with age. Also, oxidative stress increased significantly with age. Antioxidant levels, however, decreased significantly with age. Structural changes of the submandibular gland accompanying aging included atrophy of parenchyma cells and fatty degeneration and fibrosis of stroma, and the submandibular gland weight ratio decreased. These results suggest that oxidative stress increases with age, not just systemically but also locally in the submandibular gland, and that oxidative stress causes changes in the structure of the salivary gland and is involved in hyposalivation.

## Introduction

Saliva plays an important role in oral functions such as swallowing, mastication, and pronunciation, in addition to performing anti-caries and anti-fungal functions and providing physical and immunological protection to the mucous membranes of the mouth and alimentary canal.^(1,2)^ Consequently, quantitative or qualitative changes in the saliva not only cause dryness of the oral mucous membranes and discomfort, but can also cause gingivitis, caries, and myotic infection.^(3)^ In addition, hyposalivation and dry mouth, which result from dysfunction of the salivary glands, have been implicated in reduced quality of life.^(4–6)^

Generally speaking, there is often a close connection between salivary flow rate and dry mouth. Xerostomia refers to the subjective sensation of dry mouth, although it is often linked to reduced saliva production or changes in the chemical composition of the saliva. For this reason, xerostomia patients commonly have decreased salivary flow rate, particularly during rest.^(1)^ Hyposalivation, on the other hand, is strictly defined as an objective decrease in the salivary flow rate^(7–9)^ involving physiological atrophy of the salivary gland, which manifests itself as morphohistological changes in the gland.^(10,11)^

Few reports have been issued on age related morphometric changes in animal.^(12–15)^ In these studies, histologic examinations demonstrated that during aging, the parenchyma of salivary glands is gradually replaced by fat, fibrous connective tissue, oncocytes, and apoptotic salivary epithelial cells.^(12–15)^ However, the relationship between age and salivary flow rates is controversial. Age-related salivary glands showed that salivary flow rate in elderly individuals were lower than that in young individuals.^(15–17)^ On the other hand, some studies show that aging does not diminish the ability of salivary glands to produce saliva.^(18,19)^ Therefore, the mechanism between age-related structural changes of the salivary glands and hyposalivation are not clearly known.

Many studies have begun implicate oxidative stress as one of the causes of aging, age-related disease, and lifestyle disease.^(20–24)^ Reactive oxygen species (ROS), the main cause of oxidative stress, play an important role in cell signaling and metabolic processes but also contribute to pathogenic processes in variety of inflammatory disorders.^(25)^ It has also been reported that oxidative damage to the periodontal tissue by ROS is involved in the onset of periodontal disease.^(26)^ ROS and other free radicals are generally eliminated by the body’s antioxidant action, the oxidation-reduction (redox) regulatory mechanism, so that a buildup of ROS within the body is suppressed.^(23)^ However, antioxidant action decreases with age, so that if the redox balance is disrupted, oxidative damage of target molecules within the body occurs, resulting in effects such as hyperoxidation of lipids, protein denaturation, oxidative DNA damage, telomere shortening, and apoptosis.^(21,27)^ Thus, oxidative damage will also likely manifest itself in the salivary gland tissue of the elderly and will affect the saliva secretory function of the glands. However, the relationship among age-related hyposalivation and oxidative stress has not been clarified.

Here, the stimulated whole saliva flow rate, redox biomarkers, submandibular gland weight ratio, and structural changes in the submandibular gland were investigated in naturally aging mice to clarify the effects of aging and the associated increase in oxidative stress on saliva secretory function and the tissue structure of the salivary glands.

## Methods

### Naturally aging mice

The naturally aging mice were 7-week-old (7 w) male ICR mice (CLEA Japan, Inc., Tokyo, Japan) with a body weight of 35 g that were reared to 24 weeks (24 w), 30 weeks (30 w), 48 weeks (48 w), and 72 weeks (72 w) (*n* = 5 each). All animals were reared in the Bioscience Facility of the Research Center for Odontology, the Nippon Dental University School of Life Dentistry at Tokyo at a temperature of 23 ± 1°C, 50 ± 10% humidity, and a 12-h cycle of light and darkness. They had free access to food (CE-2, CLEA Japan, Inc.) and ultra-filtered water.

The handling and treatment of experimental animals was approved by the Animal Experiments Committee of the Nippon Dental University School of Life Dentistry at Tokyo and complied with the regulations regarding animal care and management.

### Sample collection

To collect samples, mice were given general anesthetic by intraperitoneal injection (40 mg/kg body weight) of 50 mg/ml pentobarbital sodium (Nembutal^®^ injection, Dainippon Sumitomo Pharma Co., Ltd., Osaka, Japan) and secured in the supine position. Then, Intraperitoneal injection (0.5 mg/kg body weight) of pilocarpine hydrochloride (Wako Pure Chemical Industries, Ltd., Osaka, Japan) was administered, and the mouse was allowed to rest for 5 min. The flow of whole saliva was sampled by absorbing saliva onto a surgical sponge (BD Visispear^TM^ Eye Sponge 7 cm, Becton Dickinson and Company, Franklin Lakes, NJ) inserted beneath the tongue for 5 min. Next, stimulated whole saliva was collected by sucking it from the mouth using a 1.0-ml syringe (Terumo Corporation, Tokyo, Japan), transferred to 1.5-ml microtubes (K.K. Ashisuto, Tokyo, Japan), and stored at −80°C. Blood (10 ml) was collected from the mouse tail vein, and serum was obtained by centrifugation (4°C, 10,000 × *g*, 20 min) and stored at −80°C until measurements were taken.

To collect submandibular gland, Infiltration anesthesia of 0.3 ml of 2% lidocaine hydrochloride containing 1/800,000 epinephrine (Xylocaine^®^ cartridge, Densply Sankin, Tokyo, Japan) was administered subcutaneously to the median cervical region, and an incision was made in the skin. The submandibular glands were separated from the surrounding soft tissue along the membrane of the glands, and the left and right submandibular glands were removed by cutting the glands at the junction of the proximal duct.

### Stimulated whole salivary flow rate

The weight of the surgical sponge and absorbed whole saliva was measured using an electric balance. The weight of the surgical sponge alone prior to saliva sampling was subtracted, and the resulting value was taken to be the stimulated whole salivary flow rate.

### Measurement of submandibular gland weight ratio

The membrane of the extracted submandibular glands and fluid sticking to the surface of the glands were removed. The left and right submandibular glands were then weighed separately on an electric balance. The average value was divided by the body weight to give the submandibular gland weight ratio.

### Oxidative damage to DNA

Quantitative measurement of oxidative damage to DNA was made using 8-hydroxy-deoxyguanosine (8-OHdG). Cryopreserved saliva was thawed at room temperature and centrifuged (10,000 × *g*, for 45 min at 4°C). The supernatant was transferred to an ultrafiltration filter (Microcon YM-10, Millipore, MA), centrifuged again (10,000 × *g*, for 50 min at 4°C), and then ultrafiltered to give the saliva sample. Cryopreserved serum was thawed at room temperature and transferred to an ultrafiltration filter in the same way, and then centrifuged (10,000 × *g*, for 50 min at 4°C) to give the serum sample. Saliva and serum samples were diluted two-fold using PBS, and 8-OHdG was measured with a microplate reader (Infinite M200) at 405 nm at room temperature using the 8-OHdG Check ELISA Kit (Japan Institute for the Control of Aging, Nikken Seil Co., Ltd., Shizuoka, Japan).

### Measurement of oxidative stress

Oxidative stress (oxidant concentration) was measured with the Free Radical Elective Evaluator (FREE, Diacron, Grosseto, Italy) using the Diacron-Reactive Oxygen Metabolites (d-ROMs) test (Diacron). Saliva and serum samples were ultrafiltered before measurement using the same protocol as for 8-OHdG.

### Measurement of antioxidant levels

Antioxidant levels (amount of ferric ion reduction) were measured with a FREE, in the same way as the d-ROMs test, using the Biological Antioxidative Potential (BAP) test (Diacron).

### Histopathological observation

The extracted submandibular glands were fixed by immersion in 4% paraformaldehyde for 24 h and paraffin embedded according to the conventional protocol. The embedded specimens were sliced to a thickness of 5 µm using a rotary microtome (HM335E, MICROM International GmbH, Walldorf, Germany), and hematoxylin-eosin (H&E) stained.

### Fluorescent immunohistochemical observation

Paraffin-embedded samples of 5-µm thickness were prepared as for H-E staining. Antigen retrieval was carried out using HistoVT One (Nacalai Tesque, Kyoto, Japan) at pH 7.0, 70°C for 20 min; blocking was carried out using 1% bovine serum albumin (Vector Laboratories, Inc., CA) at room temperature for 60 min. The samples were subsequently reacted with the primary antibody overnight at 4°C. They were then reacted with fluorescence-labeled secondary antibody in the dark at room temperature for 60 min, Proliferating cell nuclear antigen (PCNA) as proliferation maker, TdT-mediated dUTP nick end labeling (TUNEL) as apotosis maker and 8-hydroxy-2'-deoxyduanosine (8-OHdG) as oxidative maker, nuclear stained (ProLong^®^ Gold antifade reagent with DAPI, Molecular Probes^TM^, OR,), and mounted. Fluorescent immunohistochemical observation was carried out using a fluorescence microscope (IX71, Olympus, Tokyo, Japan).

### Statistical analysis

Measurements from each section were displayed as the mean ± SD. Significant differences were tested with one-way analysis of variance (ANOVA), which was tested with Tukey’s honestly significant difference (HSD) test. The statistical significance level was set at 5% (*p*<0.05).

## Results

### Stimulated whole salivary flow rate

**Fig. 1:**
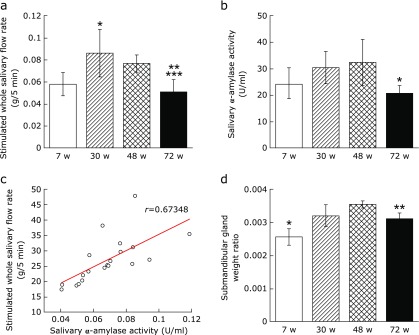
Comparison of salivary flow rate and submandibular gland weight ratio. (a) Stimulated whole salivary flow rate (g/5 min). Values are expressed as mean ± SD (*n* = 5; *p<0.05: 7 w vs 30 w, **p<0.01: 30 w vs 72 w, ***p<0.05: 48 w vs 72 w, ANOVA with Tukey test). (b) Salivary α-amylase activity (U/ml). Values are expressed as mean ± SD (*n* = 5; *p<0.05: 48 w vs 72 w, ANOVA with Tukey test). (c) Correlation between the stimulated whole salivary flow rate and salivary α-amylase activity (*r* = 0.6382). (d) Submandibular gland weight ratio. Values are expressed as mean ± SD (*n* = 5; *p<0.01: 7 w vs 30 w, 48 w, 72 w, **p<0.05: 48 w vs 72 w, ANOVA with Tukey test).

The stimulated whole salivary flow rate increased significantly from 7 w to 30 w, and then decreased significantly over time from 30 w to 72 w and from 48 w to 72 w (Fig. 1a).

### Submandibular gland weight ratio

The submandibular gland weight ratios at 30 w and 48 w were significantly greater than at 7 w, but decreased significantly from 48 w to 72 w (Fig. 1b).

### Saliva and serum 8-OHdG

**Fig. 2:**
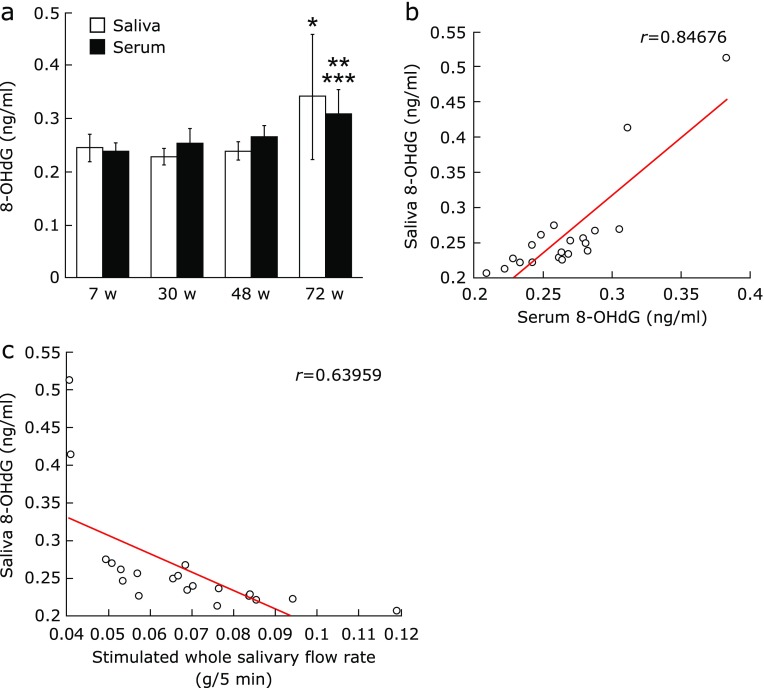
Comparison of saliva and serum 8-OHdG levels. (a) Saliva and serum 8-OHdG levels (ng/ml). 8-OHdG levels were measured using ELISA. Values are expressed as mean ± SD, (*n* = 5; *p<0.05: 30 w vs 72 w, **p<0.01: 7 w vs 72 w, ***p<0.05: 30 w vs 72 w, ANOVA with Tukey test). (b) Correlation of 8-OHdG between saliva and serum (*r* = 0.84676). (c) Correlation between the stimulated whole salivary flow rate and saliva 8-OHdG (*r* = 0.63402).

No significant differences were found in saliva or serum 8-OHdG from 7 w to 48 w. However, saliva 8-OHdG increased significantly from 30 w to 72 w, and serum 8-OHdG increased significantly from 7 w to 72 w and from 30 w to 72 w (Fig. 2a). A positive correlation was found between saliva 8-OHdG and serum 8-OHdG (*r* = 0.84676) (Fig. 2b). And, a negative correlation was found between the stimulated whole salivary flow rate and saliva 8-OHdG (*r* = 0.63959) (Fig. 2c) and between the stimulated whole salivary flow rate and serum 8-OHdG (*r* = 0.62206; data not shown).

### Saliva and serum d-ROMs test

**Fig. 3:**
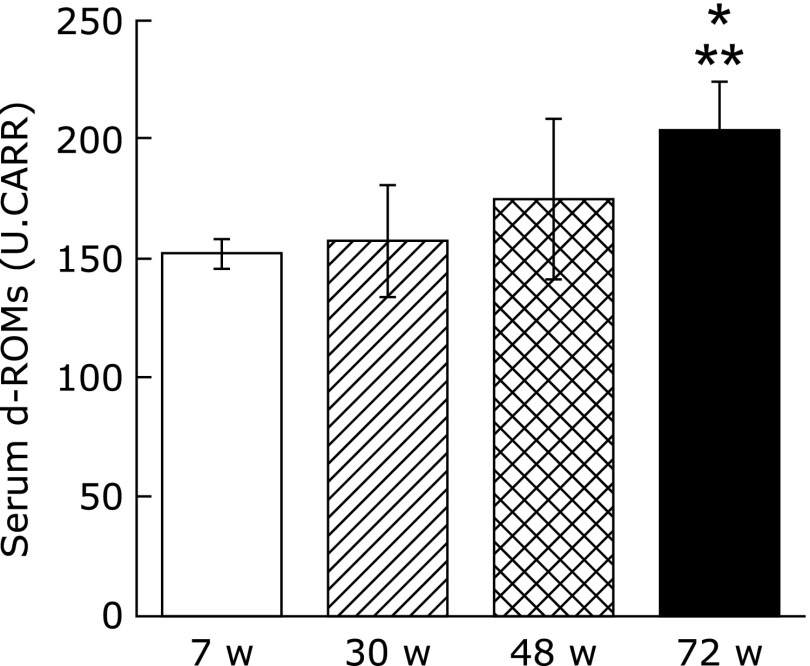
Comparison of serum d-ROMs test (U.CARR). Values are expressed as mean ± SD (*n* = 5; *p<0.05: 7 w vs 72 w, **p<0.01: 30 w vs 72 w, ANOVA with Tukey test).

For the d-ROMs test using saliva, all the model animals were below the measurement limit value. For the serum d-ROMs test, however, a significant increase was found between 7 w and 72 w and between 30 w and 72 w (Fig. 3). A negative correlation was found between the stimulated whole salivary flow rate and the serum d-ROMs test (*r* = 0.61813; data not shown).

### Saliva and serum BAP test

**Fig. 4:**
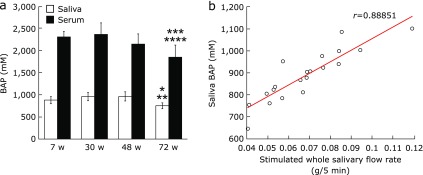
Comparison of Saliva BAP test (µMs). Values are expressed as mean ± SD (*n* = 5; *p<0.01: 30 w vs 72 w, **p<0.01: 48 w vs 72 w, ***p<0.05: 7 w vs 72 w, ****p<0.01: 30 w vs 72 w, ANOVA with Tukey test).

The saliva BAP test showed a significant decrease from 30 w to 72 w and from 48 w to 72 w, and the serum BAP test showed a significant decrease from 7 w to 72 w and from 30 w to 72 w (Fig. 4a). A positive correlation was found between the saliva BAP test value and the serum BAP test value (*r* = 0.67165; data not shown). In addition, the stimulated whole salivary flow rate was positively correlated with the saliva BAP test value (*r* = 0.88851) (Fig. 4b) and the serum BAP test value (*r* = 0.74803; data not shown).

### Histopathological examination using H-E staining

**Fig. 5:**
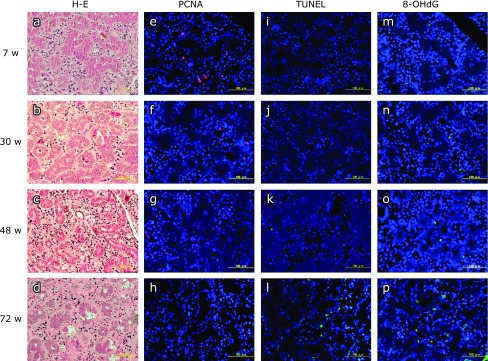
H-E and immunofluorescence staining of submandibular gland. (a–d) H-E staining of submandibular gland in a: 7 weeks, b: 30 weeks, c: 48 weeks, d: 72 weeks (scale bars: 100 µm). (e–h) PCNA immunofluorescence staining (red) of submandibular gland in e: 7 weeks, f: 30 weeks, g: 48 weeks, h: 72 weeks. Nuclei are counterstained with DAPI (blue) (scale bars: 100 µm). (i–l) TUNEL immunofluorescence staining (green) of submandibular gland in i: 7 weeks, j: 30 weeks, k: 48 weeks, l: 72 weeks. Nuclei are counterstained with DAPI (scale bars: 100 µm). (m–p) 8-OHdG immunofluorescence staining (green) in submandibular gland in m: 7 weeks, n: 30 weeks, o: 48 weeks, p: 72 weeks. Nuclei are counterstained with DAPI (scale bars: 100 µm).

Relatively large, immature parenchyma cells were found in the submandibular gland at 7 w (Fig. 5a). At 30 w, these had matured, and the submandibular gland showed a structure with abundant parenchyma (Fig. 5b). However, at 48 w, we observed fatty degeneration in the stroma and a tendency for parenchyma cells to atrophy (Fig. 5c). At 72 w, clear enlargement and fibrillization of the stroma accompanied the atrophy of the parenchyma cells (Fig. 5d).

### Immunofluorescence staining observations

#### PCNA

Many cells positive for PCNA were found at 7 w, but these showed a tendency to decrease over time, and they were only sparsely localized at 48 w and 72 w (Fig. 5e–h).

#### TUNEL

Almost no TUNEL-positive cells were found at 7 w and 30 w, but many positive cells were found at 72 w (Fig. 5i–l).

#### 8-OHdG

The number of cells positive for 8-OHdG at 7 w and 30 w was extremely small, but it increased over time, and at 72 w, many positive cells were found (Fig. 5m–p).

## Discussion

Clinically, changes in salivary flow rate are often found in elderly people using medication, particularly multi-drug medication, and increased age itself is considered to have little effect on changes in salivary flow rate.^(1)^ However, salivary flow rate needs to be measured over time under constant environmental conditions with no medication to assess the effects of aging itself. In the present study, therefore, changes in the salivary flow rate were examined in mice with no drug administration that were allowed to age naturally to 7 w, 30 w, 48 w and 72 w. Salivary flow was evaluated by measuring the whole salivary flow rate stimulated by pilocarpine hydrochloride, as this is a highly reproducible parameter for measuring salivary gland function.^(30,31)^ The stimulated whole salivary flow rates at 48 w and 72 w were significantly lower than at 30 w, and at 72 w, the rate was some 30% lower than at 30 w. At 30 w, the stimulated whole salivary flow rate was significantly greater than the 7-week control.

This decrease in salivary gland function with age is likely to be the result of changes in the tissue composition of the body of the gland. Fatty degeneration and fibrillization are degenerative changes that appear in the salivary gland as a result of aging alone.^(1)^ The present study also indicates that acinar cells, which comprise the glandular parenchyma, have a tendency to atrophy over time. At 48 w, fatty degeneration and fibrous connective tissue were apparent, and the proportion of stroma had increased. Fibrillization of the stroma was even clearer at 72 w. These structural changes in the tissue of the gland are consistent with the findings of Waterhouse *et al.*,^(30)^ who reported that functional parenchyma cells of the human submandibular gland are replaced by fat and fibrous tissue, and Scott,^(10)^ who reported that acinar cells in the parotid and submandibular glands decrease in proportion to the increase in fat and fibrous tissue. Furthermore, in the present study, the submandibular gland weight ratio decreased significantly from 48 w to 72 w, indicating atrophy. This indicates that the organ and interstitial cell organization changes as a result of the physiological aging that accompanies chronological aging, resulting in atrophy.^(31)^ The hyposalivation seen in Sjögren’s syndrome is caused by organ-specific apoptosis of the salivary gland epithelial cells accompanying disruption of the tissue of the gland.^(32,33)^ In the present study, histological examination using immunofluorescence staining showed a decrease in cell proliferation over time and an increase in apoptotic cells. This is consistent with a report^(34)^ showing that TUNEL-positive cells in mouse submandibular gland increase with age. Therefore, the implication is that changes in tissue structure due to aging reduce salivary gland function and are involved in the decrease in the stimulated salivary flow rate.

These structural changes in salivary gland tissue are believed to occur physiologically as a result of aging.^(10,11)^ Oxidative stress has recently been shown to disrupt cell structure, and this harmful process is a major mediator in the various diseases and aging that result from disruption of cell structure.^(24,26)^ ROS resulting from external stresses such as smoking, medicines, ultra-violet rays, or environmental factors accumulate within the body as it ages. Moreover, the body’s anti-oxidative capacity decreases with age, so that the redox regulatory mechanism no longer functions fully.^(21,35)^ This results in oxidative stress, and the oxidative damage manifests itself as a variety of pathophysiological effects on the cell, including apoptosis.^(23,27)^ In Sjögren’s syndrome, the level of 8-OHdG in the saliva may increase, and the function of the salivary gland may be disrupted by oxidative stress.^(36,37)^ In the present study, therefore, oxidative stress and antioxidant levels in the saliva and serum of aged mice were measured. The d-ROMs test used in the present study has been found in clinical studies to correlate with C-reactive protein in patients with periodontal disease, and the BAP test has been shown to be a useful redox marker.^(38)^ The d-ROMs test showed significantly greater saliva and serum 8-OHdG at 72 w than at 30 w. Also, the number of 8-OHdG positive cells in the submandibular gland increased over time. At the same time, the blood and serum BAP test showed a significantly lower level at 72 w than 30 w. Thus, the present study showed that oxidative stress with increasing age as seen by the disruption of the redox regulatory mechanism occurs not just systemically, but is also highly localized in the submandibular gland. Oxidative stress is brought on by inflammatory changes in cells, tissues or organs due to inflammatory cytokines such as interleukin (IL)-1 or IL-6, which are mediated by nuclear factor-κB.^(39,40)^ One organ in which dysfunction can be attributed to oxidative stress is the liver. In nonalcoholic steatohepatitis (NASH), liver function is impaired by fatty degeneration or by inflammatory cytokines such as IL-6, tumor necrosis factor-α, or IL-8.^(41)^ Consequently, because it is likely that the changes in salivary gland tissue structure brought on by aging are caused by the same mechanism as NASH, future studies will need to investigate changes over time in inflammatory cytokines in the salivary gland.

Recently, Kuraji *et al.*^(42)^ reported that astaxanthin (AX), which is anti-oxidant carotenoid, decreased the oxidative stress of serum and saliva in aging mice. In addition, AX controlled inflammations in the salivary glands and increased salivary flow rate. Therefore, the antioxidant therapy can be expected to prevent the hyposalivation caused by oxidative stress associated with aging.

The results of the present study suggest that the decrease in the stimulated whole salivary flow rate in aging mice is the result of changes in the tissue structure of the salivary gland caused by oxidative stress associated with aging. From a clinical perspective, however, the degree of oxidative stress associated with aging varies greatly depending on a range of environmental factors, such as medications, eating habits, exercise, and stress, so the results also suggest that salivary function varies according to the lifestyle and environmental factors that are the backdrop to aging.

## Abbreviations

ANOVA: analysis of variance
AX: astaxanthin
BAP: Biological Antioxidative Potential
DAPI: 4'-6-diamidino-2-phenylindole
d-ROMs: Diacron-Reactive Oxygen Metabolites
FREE: Free Radical Elective Evaluator
HSD: honestly significant difference
IL: interleukin
NASH: nonalcoholic steatohepatitis
8-OHdG: 8-hydroxy-deoxyguanosine
PCNA: proliferating cell nuclear antigen
ROS: reactive oxygen species
TUNEL: TdT-mediated dUTP nick end labeling

## References

[1] Gueiros LA, Soares MS, Leão JC (2009). Impact of ageing and drug consumption on oral health. Gerodontology.

[2] Otsuki T, Shimizu K, Zempo-Miyaki A, Maeda S (2016). Changes in salivary flow rate following Chlorella-derived multicomponent supplementation. J Clin Biochem Nutr.

[3] Nagler RM (2004). Salivary glands and the aging process: mechanistic aspects, health-status and medicinal efficacy monitoring. Biogerontology.

[4] Locker D, Matear D, Stephens M, Jokovic A (2002). Oral health-related quality of life of a population of medically compromised elderly people. Community Dent Health.

[5] Baker SR, Pankhurst CL, Robinson PG (2006). Utility of two oral health-related quality-of-life measures in patients with xerostomia. Community Dent Oral Epidemiol.

[6] Ikebe K, Matsuda K, Morii K (2007). Impact of dry mouth and hyposalivation on oral health-related quality of life of elderly Japanese. Oral Surg Oral Med Oral Pathol Oral Radiol Endod.

[7] Vissink A, Spijkervet FK, Van Nieuw Amerongen A (1996). Aging and saliva: a review of the literature. Spec Care Dentist.

[8] Cassolato SF, Turnbull RS (2003). Xerostomia: clinical aspects and treatment. Gerodontology.

[9] Ship JA, McCutcheon JA, Spivakovsky S, Kerr AR (2007). Safety and effectiveness of topical dry mouth products containing olive oil, betaine, and xylitol in reducing xerostomia for polypharmacy-induced dry mouth. J Oral Rehabil.

[10] Scott J (1987). Structure and function in aging human salivary glands. Gerodontology.

[11] Drummond JR, Newton JP, Abel RW (1995). Tomographic measurements of age changes in the human parotid gland. Gerodontology.

[12] Scott J (1977). Quantitative age changes in the histological structure of human submandibular salivary glands. Arch Oral Biol.

[13] Scott J, Bonder L, Baum BJ (1986). Assessment of age-related changes in the submandibular and sublingual salivary glands of the rat using stereological analysis. Arch Oral Biol.

[14] Sashima M (1986). Age-related changes of rat submandibular gland: a morphometric and ultrastructural study. J Oral Pathol.

[15] Choi JS, Park IS, Kim SK, Lim JY, Kim YM (2013). Analysis of age-related changes in the functional morphologies of salivary glands in mice. Arch Oral Biol.

[16] Bodner L, Baum BJ (1985). Characteristics of stimulated parotid grand selection in the aging rat. Mech Ageing Dev.

[17] Pedersen W, Schubert M, Izutsu K, Mersai T, Truelove E (1985). Age-dependent decreases in human submandibular gland flow rates as measured under resting and post-stimulation conditions. J Dent Res.

[18] Bodner L, Baum BJ (1984). Submandibular gland secretory function in young adult and aged rats. Comp Biochem Physiol A Comp Physiol.

[19] Gupta A, Epstein JB, Sroussi H (2006). Hyposalivation in elderly patients. J Can Dent Assoc.

[20] Halliwell B (1991). Reactive oxygen species in living systems: source, biochemistry, and role in human disease. Am J Med.

[21] Dröge W (2003). Oxidative stress and aging. Adv Exp Med Biol.

[22] Valko M, Leibfritz D, Moncol J, Cronin MT, Mazur M, Telser J (2007). Free radicals and antioxidants in normal physiological functions and human disease. Int J Biochem Cell Biol.

[23] Finkel T, Holbrook NJ (2000). Oxidants, oxidative stress and the biology of ageing. Nature.

[24] Indo HP, Yen HC, Nakanishi I (2015). A mitochondrial superoxide theory for oxidative stress diseases and aging. J Clin Biochem Nutr.

[25] McCord JM (2000). The evolution of free radicals and oxidative stress. Am J Med.

[26] Chapple IL, Matthews JB (2007). The role of reactive oxygen and antioxidant species in periodontal tissue destruction. Periodontol 2000.

[27] Balasubramanyam M, Adaikalakoteswari A, Sameermahmood Z, Mohan V (2010). Biomarkers of oxidative stress: methods and measures of oxidative DNA damage (COMET assay) and telomere shortening. Methods Mol Biol.

[28] Parvinen T, Larmas M (1982). Age dependency of stimulated salivary flow rate, pH, and lactobacillus and yeast concentrations. J Dent Res.

[29] Arneberg P, Storhaug K, Sandvik L (1989). Effect of a slow release transcutaneous scopolamine application on salivary flow, pH, buffering action, and salivary levels of Streptococcus mutans and lactobacilli. Scand J Dent Res.

[30] Waterhouse JP, Chisholm DM, Winter BB, Patel M, Yale RS (1973). Replacement of functional parenchymal cells by fat and connective tissue in human submandibular salivary glands: an age-related change. J Oral Pathol.

[31] Alison MR, McGee J, Isaacson PG, Wright NA (1992). Repair and regenerative responses. Oxford Textbook of Pathology: Volume 1 Principles of Pathology.

[32] Fox RI, Stern M, Michelson P (2000). Update in Sjögren’s syndrome. Curr Opin Rheumtol.

[33] Kong L, Ogawa N, Nakabayashi T (1997). Fas and Fas ligand expression in the salivary glands of patients with primary Sjögren’s syndrome. Arthritis Rheum.

[34] Enoki N, Kiyoshima T, Sakai T (2007). Age-dependent changes in cell proliferation and cell death in the periodontal tissue and the submandibular gland in mice: a comparison with other tissues and organs. J Mol Histol.

[35] Tolmasoff JM, Ono T, Cutler RG (1980). Superoxide dismutase: correlation with life-span and specific metabolic rate in primate species. Proc Natl Acad Sci USA.

[36] Ryo K, Yamada H, Nakagawa Y (2006). Possible involvement of oxidative stress in salivary gland of patients with Sjogren’s syndrome. Pathobiology.

[37] Pagano G, Castello G, Pallarodó FV (2013). Sjogren’s syndrome-associated oxidative stress and mitochondrial dysfunction: prospects for chemoprevention trials. Free Radic Res.

[38] D'Aiuto F, Nibali L, Parkar M, Patel K, Suvan J, Donos N (2010). Oxidative stress, systemic inflammation, and severe periodontitis. J Dent Res.

[39] Szabó C (1996). The pathophysiological role of peroxynitrite in shock, inflammation, and ischemia-reperfusion injury. Shock.

[40] Miyachi M, Matsuno T, Asano K, Mataga I (2015). Anti-inflammatory effects of astaxanthin in the human gingival keratinocyte line NDUSD-1. J Clin Biochem Nutr.

[41] Uysal S, Armutcu F, Aydogan T, Akin K, Ikizek M, Yigitoglu MR (2011). Some inflammatory cytokine levels, iron metabolism and oxidant stress markers in subjects with nonalcoholic steatohepatitis. Clin Biochem.

[42] Kuraji M, Matsuno T, Satoh T (2016). Astaxanthin affects oxidative stress and hyposalivation in aging mice. J Clin Biochem Nutr.

